# The Morphology and Phenotype of Monocyte-Macrophages When Cultured on Bionanofilms Substrates with Different Surface Relief Profiles

**DOI:** 10.3390/biom10010065

**Published:** 2019-12-30

**Authors:** Natalia G. Menzyanova, Svetlana A. Pyatina, Alexander V. Shabanov, Ivan V. Nemtsev, Dmitry P. Stolyarov, Dmitry B. Dryganov, Eugene V. Sakhnov, Ekaterina I. Shishatskaya

**Affiliations:** 1 Siberian Federal University, 79, Svobodnyav, 660041 Krasnoyarsk, Russia; Davcbetik@mail.ru (S.A.P.); shishatskaya@inbox.ru (E.I.S.); 2 L.V. Kirensky Institute of Physics, Siberian Branch of the Russian Academy of Sciences, 50/38 Akademgorodok, 660036 Krasnoyarsk, Russia; alexch_syb@mail.ru; 3 Federal Research Center Krasnoyarsk Scientific Center of the Siberian Branch of the Russian Academy of Sciences, 50 Akademgorodok, 660036 Krasnoyarsk, Russia; ivan_nemtsev@mail.ru; 4 Federal Center for Cardiovascular Surgery, 45 Karaulnaya, 660020 Krasnoyarsk, Russia; dmittriystolyarov@gmail.com (D.P.S.); skymaniac@yandex.ru (D.B.D.); sakhnovev@mail.ru (E.V.S.)

**Keywords:** atherosclerosis, monocyte-macrophages morphology, alkanoate-based bionanofilms, surface relief

## Abstract

The effect of surface relief profiles of alkanoate-based bionanofilms to the monocyte-macrophages (MN-MPhs) from peripheral blood of patients with atherosclerosis was studied in vitro. Patients were subjected to coronary stenting. Cell morphology and phenotype (expression of CD antigens, levels of production of marker cytokines) in vitro were analyzed before and after the installation of stents. It was shown, that the mean square roughness (Rq) of the bionanofilms determined the variability of cell morphology, CD antigens spectraand activity of production interleukins-6 and -10. Also, it was revealed, that the “activity” of the surface topography of biopolymer substrates depends on the functional state of MNs, isolated in different time points: Before and after stenting the ratios of cell morphotypes and production of cytokines in MN-MPhs differed significantly.

## 1. Introduction

The surface topography of scaffolds is included in the regulation of paracrine signaling of the processes of proliferation, differentiation and cellular immunity through the system of mechano-chemical transduction in vitro and in vivo [1,2,3,4]. In addition, in tissue bioengineering (TE) surface features of the scaffold may affect the biogenesis of the extracellular matrix [5].

The induced with the features of surface relief intracellular molecular rearrangements are accompanied by the formation of specific morphological cellular responses: The degree of cell spreading, cell motility and orientation, the actin morphology, the formation of lamellipodia and filopodia, and endo- and exocytose vesicles significantly vary at different surface relief profiles [6,7]. So, onto the nanopatterns of surface relief been more active cell spreading, than onto the micropatterns (this is the result of the influence of the surface relief elements on the sizes of the focal adhesion sites) [8,9].

The correlations between cell morphology and cell phenotype are currently not sufficiently well investigated. It is known that the actin morphology can influence the “choice” of the differentiation line for stem cells [10]. Also, it is well-known, fully spread cells are characterized by low proliferation activity. Integrins in focal adhesion complexes are mechanically connected to the nucleus through actin cytoskeleton, and may affect the nuclear cell cycle processes [11].

Control of cell morphology through surface features is considered as a promising approach in the targeted regulation of the processes of cell differentiation and proliferation in TE. It is assumed, that surface reliefs with the hierarchical organization may exhibit a significantly higher level of biological activity than “droningly”-organized reliefs [12].

Screening systems of biological activity are being developed for various surface topography options, based on dynamics of cells morphological parameters, for polymer materials, which may be of interest for the manufacture of medical implants [9]. In this regard, in the present work, we studied the morphological features of monocyte-macrophages (MN-MPh), membrane CD-antigenic spectrum and the expression activity of markers of M1/M2 polarization (interleukins-6 and -10) when cultivated on bionanofilms with different surface relief.

Polyhydroxyalcanoates (PHAs), polymers of hydroxy-alkanoic acids, reserved molecules of carbon and energy storage depo of microorganisms, are relatively fresh materials in tissue engineering, compared with poly-d,l-lactide/glicolide, and have good biological and processing properties, together with full biodegradability. These microbial products can be obtained in high purity and sterilized with conventional techniques, intended for polymer medical implants. Naturally synthesized PHAs can consist of some types of monomers, such as 3-hydroxybutirate, 4-hydroxybutirate, 3-hydroxyvalerate, etc. The monomer composition of PHA-samples affects the material bulk properties and surface topography of products [13].

## 2. Materials and Methods

### 2.1. Patient Selection

Patients, four men and four women, aged 60–70 years, from the Cardiology Center, Krasnoyarsk, diagnosed with coronary ischemia, were involved. Studies were performed with the permission of the Ethical Committee of the Center (Minutes No. 18, 07/14/2017). Transluminal balloon angioplasty with the 2nd generation coronary stents with everolimus (Promus Element plus, Boston Scientific Corporation, USA, or XienceXpedition, ABBOT VASCULAR, USA) was performed for all patients.

### 2.2. The Study of Biopolymer Films

#### 2.2.1. PHA-Bionanofilm Samples

To obtain bionanofilms highly purified PHA-samples of the following compositions, mass%, were used:

- Film 1: Copolymer of 3-hydroxybutyrate and of 4-hydroxybutyrate [P (3HB/4HB) 92.0/8.0].
- Film 2: Copolymer of 3-hydroxybutyrate, 3-hydroxyvalerate and 3-hydroxyhexanoate [P (3HB/3HV/3HHx) 66.4/23.4/10.2].
- Film 3: Copolymer of 3-hydroxybutyrate, 3-hydroxyvalerate, 4-hydroxybutyrate and 3-hydroxyhexanoate [P (3HB/3HV/4HB/3HHx) 63.5/19.4/12.3/4.8].
- Film 4: Copolymer of 3-hydroxybutyrate and 3-hydroxyvalerate [P (3HB/3HV) 85.0/15.0].
- Film 5: Poly-3-hydroxybutyrate (P3HB) 100.

Films were obtained by pouring 2%-solutions of PHAs in dichloromethane onto a degreased glass surface, followed by evaporation of the solvent in a closed chamber. The films were cut into discs of the appropriate size, which were placed in the wells of 96-well culture plates, sterilized by exposure to 70% ethanol and UV radiation.

#### 2.2.2. Atomic Force Microscopy

Peculiarities of surface relief of films were analyzed by an atomic force microscope (multimode scanning probe microscope Solver P-47, NT-MDT, Zelenograd, Russia). The average roughness (Ra) and the mean square roughness (Rq) were calculated in 10 points as the arithmetic average of the absolute values of the height variations, 5 the highest and 5 the deepest points of the mean profile line, using standard equations. Sections of 20 × 20 μm were investigated, then 2 × 2 μm areas of local maxima and minima were selected and analyzed with a higher resolution re-analysis, and the roughness of each sample was calculated, as the average of 3 measurements.

### 2.3. Isolation and Cultivation of Monocytes

Monocytes (MNs) were isolated in hypertonic density gradient of Ficoll-verografin, according to the Recalde H. [14]. The isolated cells were suspended in DMEM (Thermo Fisher Scientific, Waltham, MA, USA) with 10% fetal serum (Thermo Fisher Scientific, Waltham, MA, USA) and the cell concentration was adjusted to 2 × 10^6^ cells/mL. In 96-well culture plates (Sigma-Aldrich, St. Louis, MO, USA) disks of bionanofilms were placed on the wells bottom and 100μL of DMEM medium and 50μLof cell suspension per well were added (10^5^ cells in each well). A culture plastic (CP) served as a control. Cells were cultured in a CO_2_-incubator (SANYO Electric Co., Ltd., Osaka, Japan) for 6 days, whereupon cultural filtrates were collected. In cultural filtrates, the content of interleukin-6 (IL-6) and interleukin-10 (IL-10) was determined by enzyme immunoassay (Human IL-10, Human IL-6 ELISA Kit, Biolegend, San Diego, CA, USA).

### 2.4. Antigenic Spectrum of the MN Population

Antigenic phenotypes of MNs were determined by flow cytometry, using direct immunofluorescence. Fixed MNs were washed with phosphate buffer, and the cell concentration was adjusted to 10^6^/mL. MNs were incubated with monoclonal antibodies (Abcam, GB) for 40 min at room temperature. The following panels were used: CD14-CD16; CD14-CD163; CD14-CD68; CD62 L-CD31; CD206-CD68; CD36-CD31. Analysis of stained cells was performed on a Navios flow cytometer (Beckman Coulter, Brea, CA, USA).

### 2.5. Electron Microscopy

On day 6, the culture medium was removed from the wells, and cells were fixed with 2.5% glutaraldehyde (Panreac, Barcelona, Spain) and with 0.1% OsO_4_ (Sigma-Aldrich, St. Louis, MO, USA). After washing, cell samples were passed through a battery of alcohols of increasing concentration. Samples were sprayed with gold in magnetron EmitechK575XD (Quorum Technologies, Lewis, UK), and analyzed using a TM 3000 microscope (HITACHI, Tokyo, Japan).

## 3. Results

### 3.1. 3-D Surface Topography of PHA Bionanofilm Samples

All five variants of polymers were characterized by well-developed surface relief. Some features of the surface relief could be observed with a small magnification using a TM 3000 microscope; a thin 3D-relief image was reconstructed using AFM.

**Figure 1:**
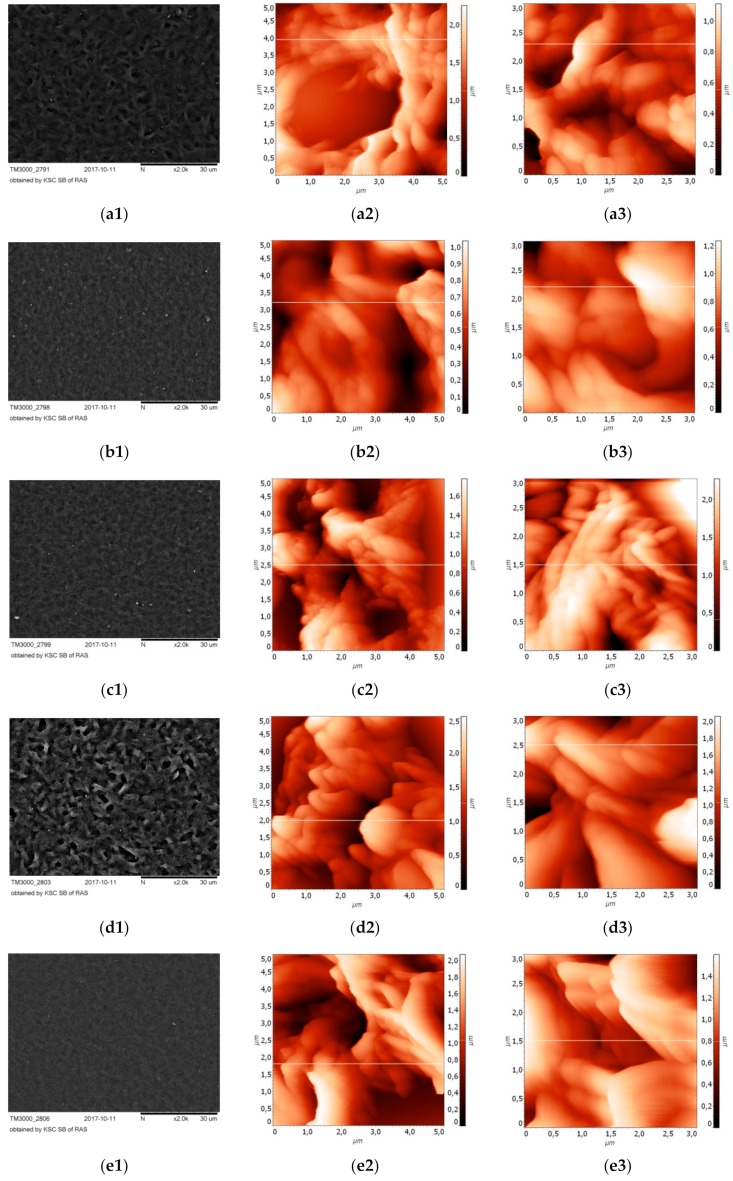
Surface topography of polyhydroxyalcanoates (PHA)bionanofilms. (**a1**–**e1**)—surface topography of films 1–5, scanning microscopy; 3-D reconstructions of surface reliefs: (**a2,a3**–**e2,e3**)—Films 1–5 (surface areas 5 µm × 5 µm, 3 µm × 3 µm), AFM.

The surface relief of sample 1 was represented by short, loose and randomly arranged fibrils, of 1–2 μm thick [Figure 1(a1)]. On the surface of the fibrils, a relief was reconstructed, in which “soft” folded structures were combined with small spherical formations [Figure 1(a2,a3)]. Among the fibrils, there were irregularly shaped pores with a diameter of 3–4 μm. The root mean square roughness of the surface relief, Rq, for film 1 was 1180 ± 212 nm.

The surface relief of sample 2 resembled small ripples on the surface of the water [Figure 1(b1)]. Small, indefinitely shaped invaginations were combined with elevations, on which either thin crest-like outgrowths were located [Figure 1(b2)], or disordered, soft shallow folds [Figure 1(b3)]. Rq for this film 2 was 993 ± 125 nm.

The surface relief of sample 3 at a slight increase showed significant similarity with the relief of sample 2, but 3D reconstruction revealed significant differences between these samples. On the surface, crater-like formations were revealed, [Figure 1(c2)], the walls of the craters were formed by folded and fine-grained structures. In the surface relief of sample 3 characteristic folded flows [(Figure 1(c3)]. Rq was 1351 ± 218 nm.

The surface relief of sample 4 had a loose, layered structure with numerous, irregularly located pores of various shapes [Figure 1(d1)]. The thin relief was represented by numerous spiky outgrowths, tightly pressed against each other [Figure 1(d2)], as well as pointed lamellar structures [Figure 1(d3)]. Rq for this film was 1219 ± 154 nm.

The surface relief of sample 5 was characterized by numerous small pores and fine-grained ripples [Figure 1(e1)]. This granularity was due to the presence of small spines on the surface of the “rigid” comb structures. Rq was 1039 ±176 nm.

It should be noted, that nano-dimensional surface profiles of thin casting films is a variable parameter, sensitive to air temperature during evaporation, atmospheric pressure, type of solvent and its purity, what determines the kinetics of crystallization processes of polymers.

A characteristic feature of the surface reliefs of films 1, 2, and 3 was realized in the presence of soft, rounded structures. Pointed and spiky structures were revealed in the surface relief of films 4 and 5. AFM 3D-reconstruction indicates a complex hierarchical organization of surface reliefs of copolymer polyhydroxyalkanoates, and the formation of characteristic recognizable combinations of relief micro- and nanopatterns for different monomers in it.

### 3.2. Expression of Marker CD Antigens on MNs, Isolated from Blood before and after Stenting

Using a gradient of hypertonic ficoll-verografin allows to isolate MN fraction with a high purity: In the selected cell population before and after stenting 96–99% of the cells expressed CD14—the main marker, which is used for sorting of MNs. Before and after stenting, the CD14^+^CD16^−^-phenotype, the classic MNs dominated among CD14^+^-MNs (95 ± 5% of cells). MNs with CD14^low^CD16^+^- phenotype (non-classical MNs) were not identified.

In addition to the main marker antigens, several complementary CD antigens were detected to analyze the heterogeneity of the CD14^+^-MN population. The functions of the selected CD antigens are given in Table 1. To characterize cellular phenotypes, pairs of CD-antigens and 4 possible variants of their expression for each pair were used:

(1) CD62L and CD31,

Options of expression: CD62L^−^CD31^+^; CD62L^+^CD31^+^; CD62L^−^CD31^−^; CD62L^+^CD31^−^;

(2) CD206 and CD68,

Options of expression: CD206^−^CD68^+^; CD206^+^CD68^+^; CD206^−^CD68^−^; CD206^+^CD68^−^;

(3) CD36 and CD31,

Options of expression: CD36^−^CD31^+^; CD36^+^CD31^+^; CD36^−^CD31^−^; CD36^+^CD31^−^.

**Figure 2:**
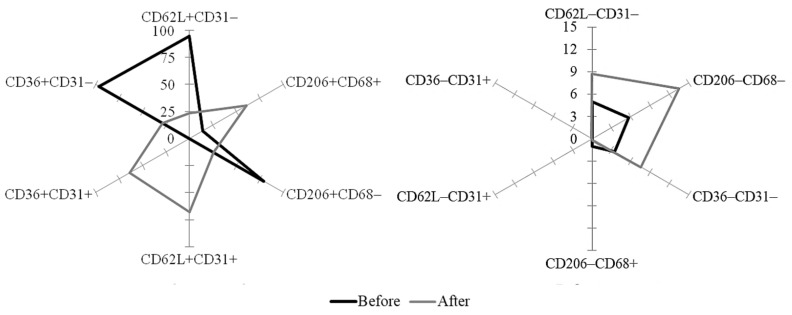
The ratio of CDphenotypes of MNs isolated from patients’ blood before stenting (Before) and after stenting (After). The relative abundance of CD phenotypes is expressed as a percentage. The abundance of 3 CD phenotypes, CD36^−^CD31^+^, CD62^−^CD31^+^, CD206^−^CD68^+^, before stenting and after stenting did not differ significantly. For each of the remaining 9 CD phenotypes, were revealed statistically reliable differences in Before and After variants (*p* < 0.05).

The number of cells, expressing a particular variant of CD antigens, was expressed as % of all CD14^+^-MNs. Variants of expression of CD antigen pairs were considered as variants of CD phenotypes of MNs.CD62L^−^CD31^+^ and CD36^−^CD31^+^-phenotypes in the CD14^+^-MN population, isolated before stenting, were not detected. Before stenting CD62L^+^CD31^−^ (94.9 ± 4.3%), CD206^+^CD68^−^ (79.1 ± 12.3%) and CD36^+^CD31^−^ (96.3 ± 3.5%) phenotypes were the most numerous (Figure 2).

After stenting, the relative abundance of various CD phenotypes in the population of isolated MNs changed significantly. A sharp decrease in the number of CD phenotypes, dominated before stenting was observed: Number CD62L^+^CD31^−^, CD206^+^CD68^−^ and CD36^+^CD31^−^ phenotypes diminishing in 4.3 and 3.4 fold, respectively. After the stenting, were the most numerous CD62L^+^CD31^+^ (67.8 ± 12.4%), CD206^+^CD68^+^ (60.9 ± 10.5%) and CD36^+^CD31^+^ (63.8 ± 11.3%) phenotypes (Figure 2). Thus, the stenting procedure led to a significant increase in the number of MNs, expressing CD31^+^ and CD68^+^.

At the next stage of our research, we studied the influence of the quantitative ratios of CD phenotypes to behavior of MN-MPhs in culture: Morphology, cell motility and activity of interleukins production.

### 3.3. The Morphology of MN-MPhs on PHA Bionanofilm Samples with Different Surface Reliefs

**Figure 3:**
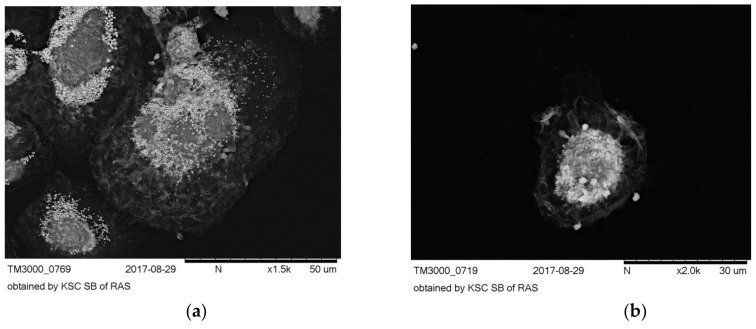

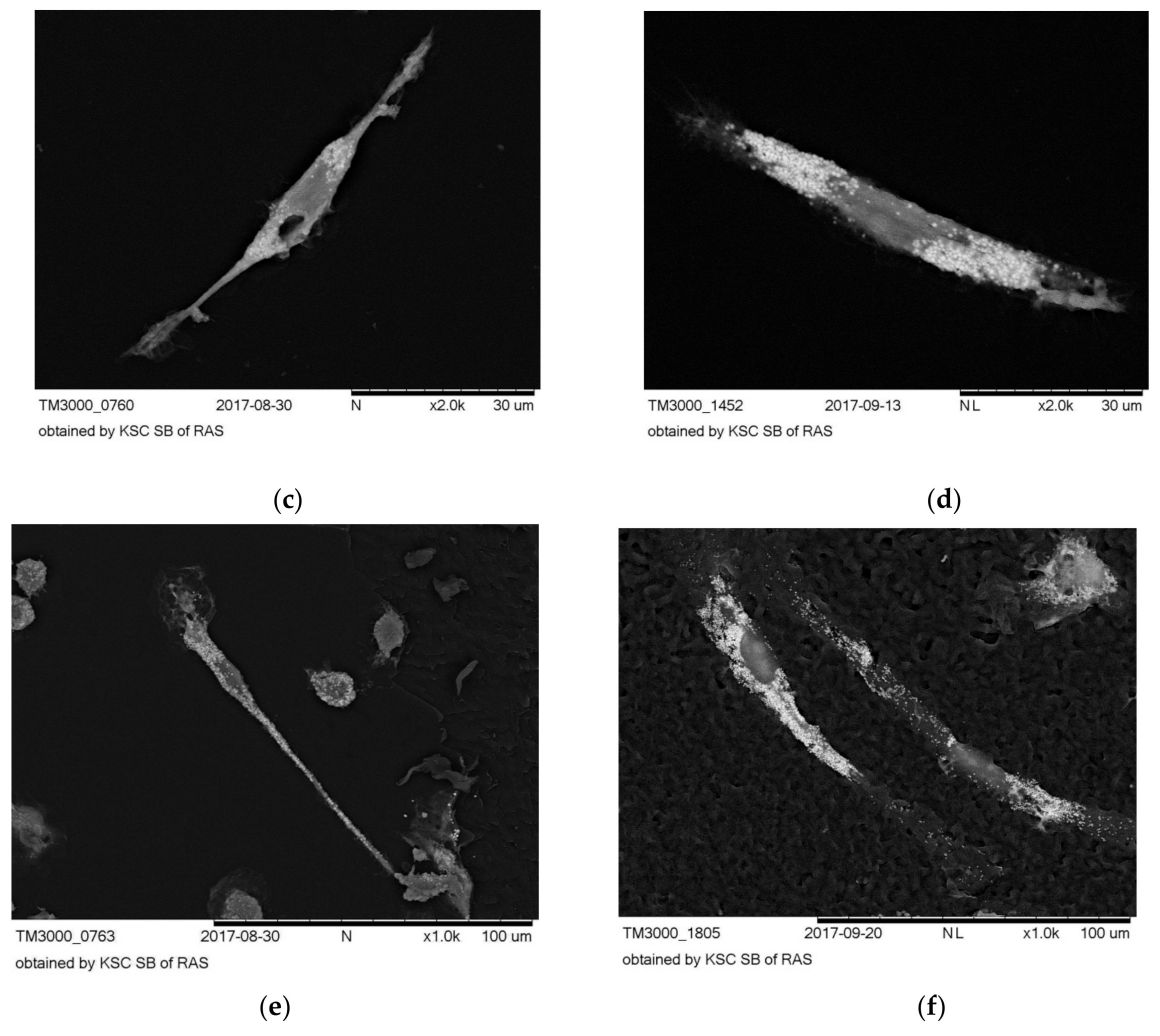
The main morphological classes (MC) of MPhs. Cultivation for six days in vitro. (**a**,**b**)—rounded cells, MC1; (**a**)—morphotype of multinucleated cells, (**b**)—morphotype of mononucleated cells. (**c**–**e**)—elongated cells, MC2. (**c**) –morphotype of spindle-like cells; relatively “short” cells with a pronounced central fusiform thickening of the body. (**d**)—morphotype of rod-shaped cells; elongated cells of approximately same diameters along the entire length. (**e**,**f**)—morphotype of filiform cells; very long, “thin” cells, the cell length varies from 80 µm to 150 µm, in this cell morphotype elongated, ovoid-shaped nuclei were observed. For (**a**–**d**) ×2000, for (**e**,**f**) ×1000.

Based on the morphological analysis, two main morphological classes of MPhs were identified in culture: Morphological class 1 (MC1)—rounded cells, and morphological class 2 (MC2)—elongated cells (Figure 3). Both morphological classes were present during the cultivation on all types of polymer films and on cultural plastic. 

The abundance ratio of these two morphological classes, MC1/MC2, varied significantly on films with different surface relief. So, before stenting, the ratio of MC1/MC2 was the highest on film 2 (2.32), and the lowest on sample 1 (0.57). After stenting, the ratio of MC1/MC2 changed significantly for each substrate. So, for films 1 and 5, the ratio of MC1/MC2 increased, i.e., after stenting, the relative number of round-shaped MPhs increased. For films 2, 3, and 4, the MC1/MC2 ratio decreased, i.e., after stenting, the number of elongated MPhs increased. It should be noted that on culture plastics, the ratio of MC1/MC2 was the same for the variant before and after stenting. 

Within each of two morphological classes, several morphotypes were distinguished. In MC1, cells of two morphotypes were observed: Rounded multinucleated MPhs (MC1I, 1st morphotype) and rounded, mononucleated MPhs (MC1II, 2nd morphotype) (Figure 3a,b). The relative abundance of MPhs of MC1I and MC1II morphotypes varied significantly on samples prior to stenting. After stenting on films 2, 3, 4 and 5, the numbers of morphotypes changed significantly also. So, on sample 2 after the stenting, the number of mononucleated MPhs decreased in 5.7 times, compared with the probe, harvested before surgery. After the stenting, the number of MC1I MPhson films 3 and 4 decreased in 2,2 and 2,7 times, respectively, and on films 5t, on the contrary, it was increased 1,6 times. (All noted differences are significant, *p* < 0.05).On culture plastic and on sample 1, the abundance of MPhs of 1st and 2nd morphotypes did not significantly differ before and after stenting.

Among the MC2 three morphotypes were distinguished, differing in the magnitude of the elongation factor (EF). The elongation factor was calculated as the ratio of the cell length to its width (at the widest point).

MC2I (Mt1) 1st morphotype, filiform cells, EF = 28.34 ± 3.22 (Figure 3e).

MC2II (Mt2) 2nd morphotype, spindle-like cells, EF = 5.39 ± 0.43 (Figure 3c);

MC2III (Mt3) 3d morphotype, rod-shaped cells, EF = 8.14 ± 0.63 (Figure 3d).

(Varieties of meanings of EF for 1st and 2nd, 1st and 3d, 2nd and 3d morphotypes were statistically significant (*p* < 0.05)).

**Figure 4:**
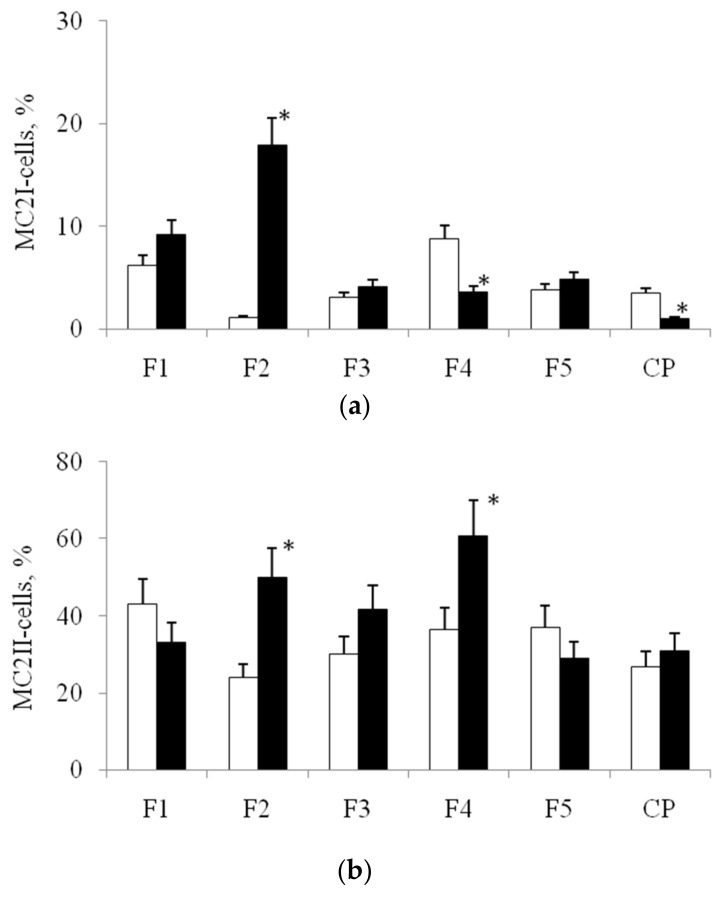

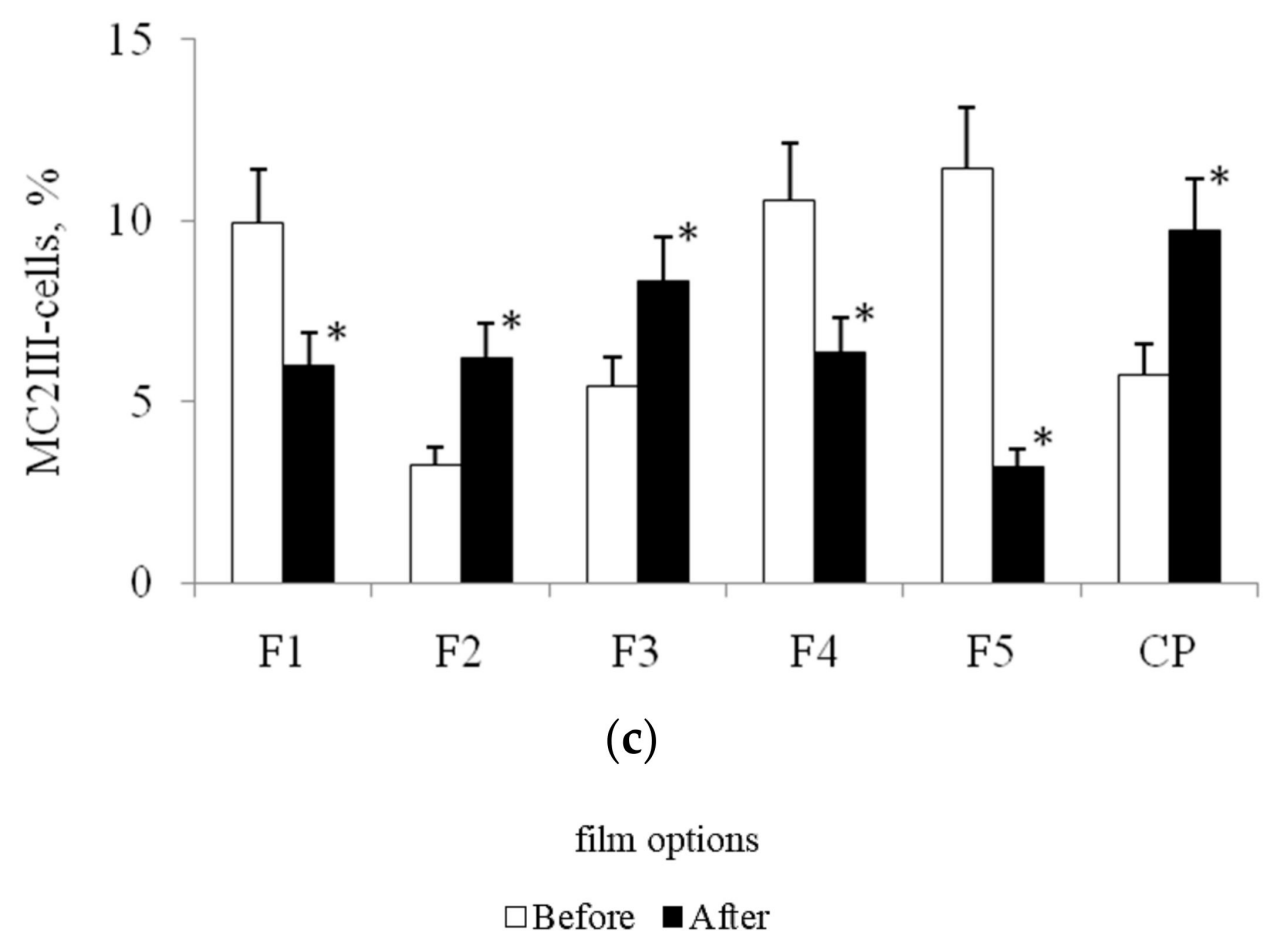
Quantity of cell by morphotypes in MC2 MPhs on PHA-samples before (Before) and after stenting (After).MC2I—filiform cells (**a**), MC2II—spindle-shaped cells (**b**), MC2III—rod-shaped cells (**c**). CP—cultural plastic. F1–F5—PHAs-films. *—values for the variant after stenting, significantly different from the variant before stenting (*p* < 0.05).

The morphotype of spindle-like cells MC2II MPhs was dominant in all variants of cultivation; however, for each type of substrates, specific quantitative relationships between these three morphotypes were formed, before and after stenting. For example, on film 2, the number of filiform cellsMC2Iafter the stenting was increased in 18 times, but on the film 1—only in 1,5 fold, compared with the meaning before stenting. The number of filiformcells MC2I MPhs after stenting decreased on film 4 and on culture plastic in 2,4 and 3,5 times, respectively, compared with the option before stenting (Figure 4a). A significant increase in the number of spindle-like MC2II MPhs cells was observed after stenting on films 2 and 4 (Figure 4b). After stenting, significant changes in the abundance of the rod-shaped cell morphotype MC2III were observed for all variants of PHA-films and for culture plastic, as compared with the variant before stenting (Figure 4c).

**Figure 5:**
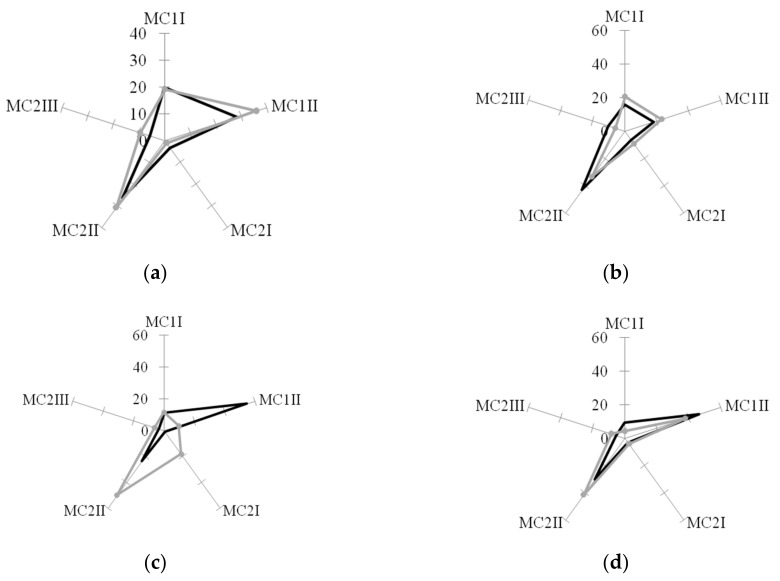

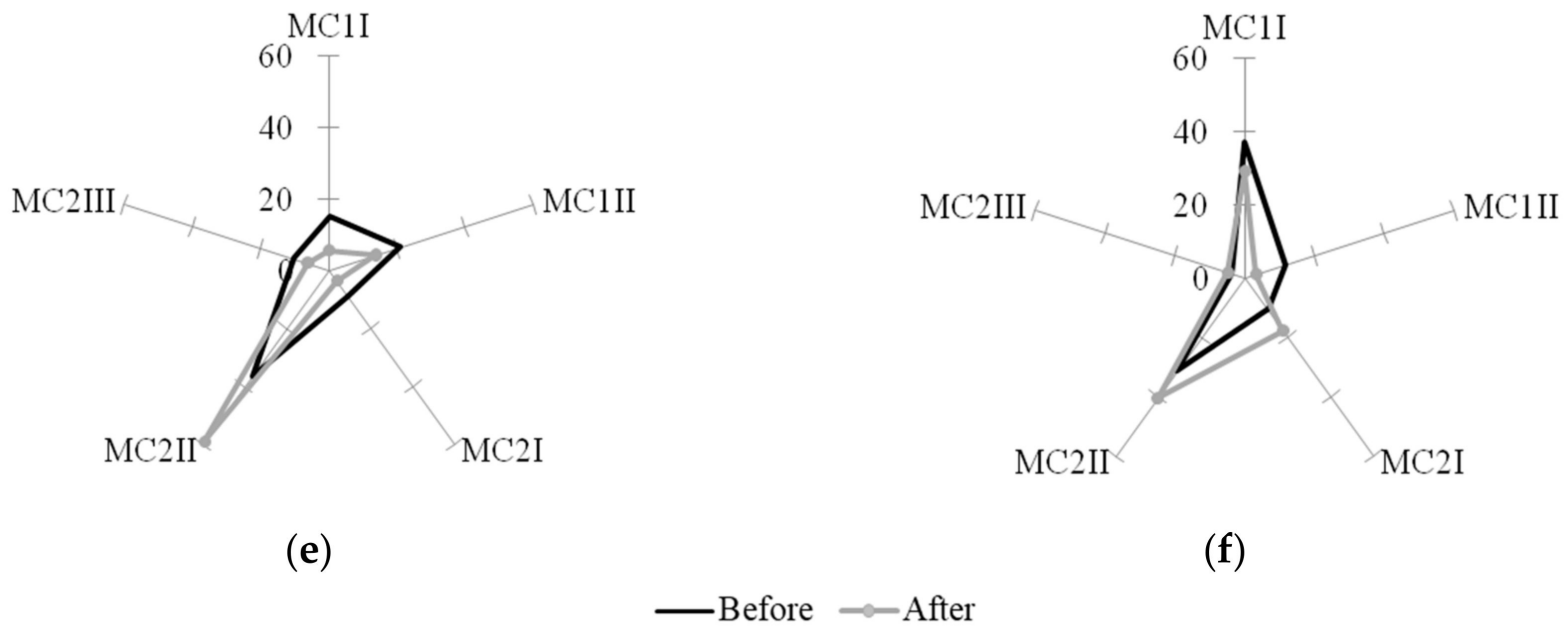
Quantity of cell in both morphological classes, MC1 and MC2, on PHA-films films with different surface relief before (Before) and after (After) stenting. MC2I—filiform cells, MC2II—spindle-shaped cells, MC2III—rod-shaped cells, MC1I—multinuclear rounded cells, MC1II—mononuclear rounded cells. (**a**) cultural plastic, (**b**–**f**)—PHAs-films, 1–5.

The obtained results indicate the influence of the features of the surface relief to the morphology of MPhs. Each relief was characterized by the formation of a specific relationship between different morphotypes of MPhs in MC1 and MC2 (Figure 5). Moreover, the morphological variability of MPhs changed significantly after the stenting: New relationships between different MPhs morphotypes formed on the same surface profiles.

The morphological variability of MPhs an in vitro culture can be associated with the processes of M1/M2 polarization. In this regard, at the next stage of work, we analyzed how the ratio MC1/MC2 was associated with the activity of the production of marker cytokines during MPh-polarization.

### 3.4. The Concentration of IL-6 and IL-10 in the MPh Culture Filtrate

**Figure 6:**
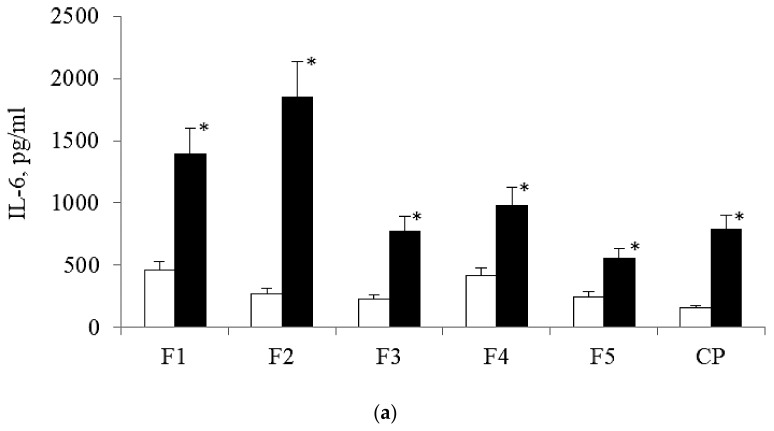

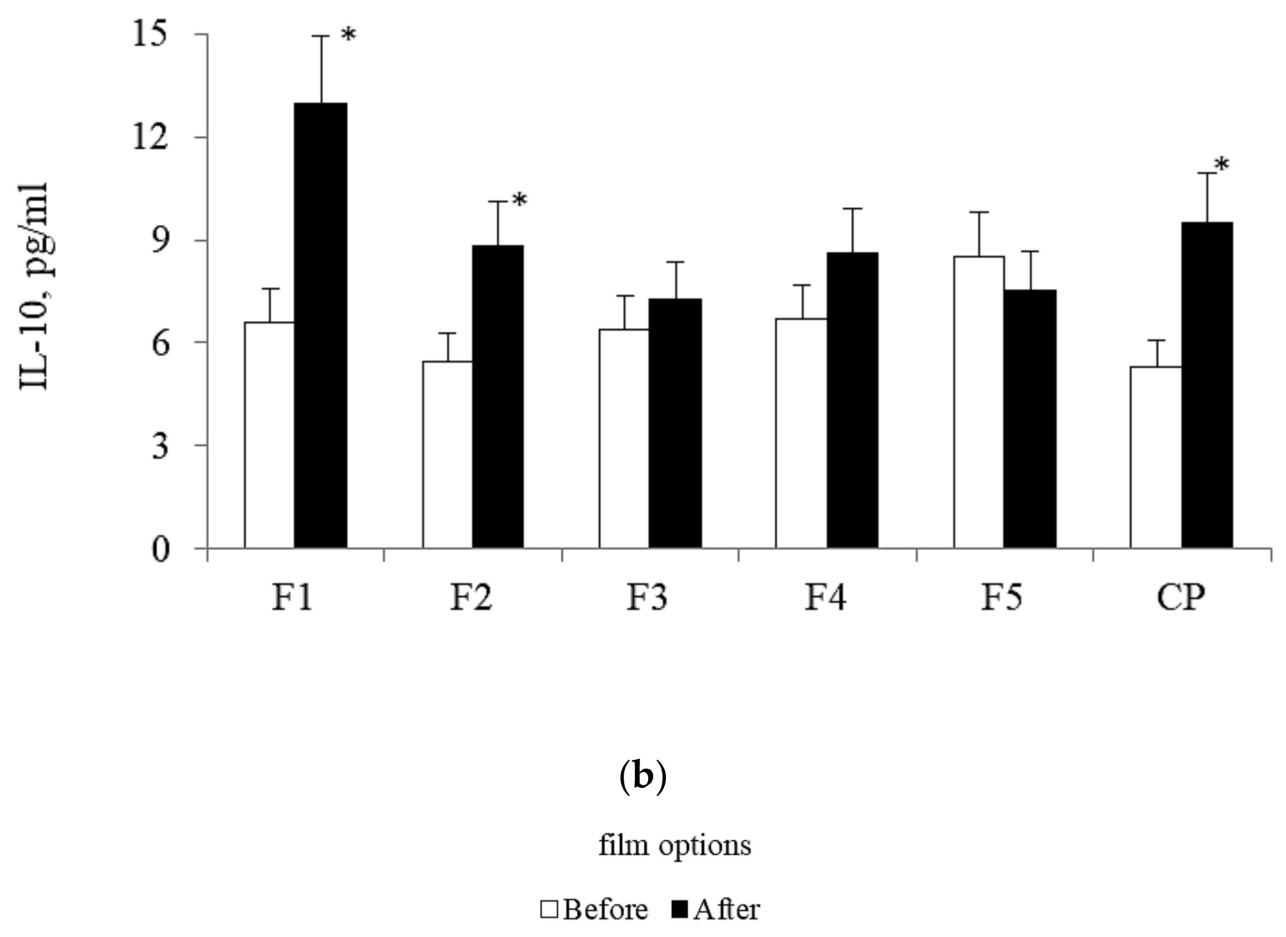
The concentration of IL-6 (**a**) and IL-10 (**b**) in the cultural filtrates on the 6th day of cultivation on PHAs-films before (Before) and after (After) stenting.CP—cultural plastic. F1–F5—PHA-films. *—The values for the variant after stenting, significantly different from the variant before stenting (*p* < 0.05).

Processes of polarization of the MPhs are accompanied by changes in levels of production of marker cytokines, in particular, IL-6 (M1-polarization) and IL-10 (M2-polarization). In the “before stenting” variant, the minimal concentration of IL-6 was founded in MPh-culture filtrates for control—cultural plastic (Figure 6a).

On samples 2, 3 and 5 the concentration of IL-6 were close and was approximately to 246.98 ± 39.16 pg/mL (1,5 times higher, than on culture plastic, *p* < 0.05), on samples 1 and 4– 396, 68 ± 60.32 pg/mL (2,4 times higher, than on cultural plastic, *p* < 0.05). The concentration of IL-10 was significantly lower, than that of IL-6 in all probes (Figure 6b). In the pre-stenting variant, the concentration of IL-10 during the cultivation in control and on samples 1, 2, 3 and 5 were the same. On film 4 the concentration of IL-10 was in 1.6 higher, than on culture plastic. After the stenting, the concentration of IL-10 was higher on sample 1and 2and on culture plastics in 2; 1.6 and 1.8 times, respectively, compared with the option before stenting (*p* < 0.05).

The results indicate, that in the 6-day-old culture of MPhs, the activity of IL-6 production was significantly higher, than the production of IL-10 before and after stenting. After the stenting, the activity of IL-6 production increased in all samples, but the activity of IL-10 production increased only on samples 2 and 5. Levels of increasing of interleukin production vary in different PHA-films. This proves the influence of PHA-surface relief features on the activity of production both, IL-6 and IL-10 in MPhs with AS.

## 4. Discussion

The population of human blood MNs is characterized by structurally functional heterogeneity. The use of two markers, CD14 and CD16, allows to reveal three subpopulations of MNs with various types of functional activity in human blood, and these data correspond with the literature: Classical CD14^++^CD16^−^-MNs, intermediate CD14^++^CD16^+^-MNs, and non-classical CD14^+^CD16^+^-MNs. Classical MNs play an important role in the initiation and progression of the inflammatory response, having pro-inflammatory roles in multiple chronic diseases, including atherosclerosis [14]. Non-classical MNs seem to play a role in the resolution of inflammation and have the ability to clear dying cells [15], viruses [16], and tumor cells from the circulation [17]. The function of the intermediate subset is mixed, with studies supporting both pro-inflammatory [18,19], and anti-inflammatory roles [20,21].

In patients with diagnosed chronic coronary ischemia, subjected to coronary stenting, only one cell type, a subpopulation of classical CD14^++^CD16^−^-MNs, was detected before stenting. An increase in the number of this subpopulation of MNs accompanies the biogenesis of atherosclerotic plaque [14]. Probably, in the late stages of chronic inflammation and destabilization of the atherosclerotic plaques CD14^++^CD16^−^-subpopulation becomes the single structurally functional phenotype of MNs.

The use of additional CD-markers allows the isolation of a significantly larger number of structurally-functional phenotypes in the population of MNs. So, Anouk et al. [22] identified eight phenotypes: Four phenotypes in a subpopulation of classical MNs, three phenotypes in a subpopulation of non-classical MNs, and in a subpopulation of intermediate MNs, only one phenotype.

In our work, to assess the structure-functional heterogeneity of the MN population, we used CD-markers, which play a key role in the implementation of MN adhesion on the endothelium, trafficking through endothelium layer, in the processes of homing and phagocytosis of modified LDL (Table 1). In the MN population, 12 phenotypic variants were distinguished, varying by the activity of expression of CD-markers in different pairs (Figure 2).

The existence of phenotypic variants of circulating MNs is associated with different stages of cell differentiation [24], which can be modified with various factors, and in particular, with surgical intervention. Thus, in the MN population, isolated before and after stenting, the relative abundance of different phenotypes altered significantly (Figure 2). Phenotypes, dominated in the MN population before stenting, were characterized by the expression of CD62L, CD36, and CD206-antigens, which are responsible for the processes of rolling, adhesion of MN to the vascular endothelium, and phagocytosis. A day passed after stenting, the number of phenotypes sharply increased, which together with CD62L, CD36 and CD206 antigens, expressed CD31 and CD68-antigens, playing a key role in endothelium trafficking, crawling over selectin bearing cells (Figure 2). The data obtained suggest a high variability of phenotypes in the population of circulating MNs. The alterations in structural and functional phenotypes of circulating MNs is observed under normal and pathological conditions [25], and has an adaptive character.

Vessel stent is a fairly rigid mechanical structure, damaging the endothelium, resulting to a local gradient of cellular degradation products, the formation of chemical signals, that may affect the MNs, passing through the stent zone.Stenting can provoke an ‘aggressive’ behavior of MNs regarding the surface of stent, and the formation of “hot spots” on it, that may interfere with the processes of stent re-endothelization, activate the proliferation of smooth muscle cells and modify dynamic of polarization processes of MPhs, and ultimately, lead to re-stenosis.

Supposedly, restructuring of the antigenphenotypes of circulating MNs is realized directly in the bloodstream. So, the use of such type of in vivo activated MNs may be perspective for the screening of materials for medical purposes. Thus, at the next stage research the behavior of MNs, harvested before and after stenting, were studied with the culturing on different PHA-bionanofilms.

Differentiation of a heterogeneous population of MNs in vitro on various PHA-bionanofilms led to the appearance of a morphologically heterogeneous population of MPhs. Various morphological forms of MPhs were grouped into two main morphological classes (MC): MC1—rounded cells, MC2—elongated cells.In the MC1 two morphotypes were distinguished: Rounded multinucleated MPhs (MC1I) and rounded mononucleated MPhs (MC1II). In the MC2, three morphotypes were observed: Filiform MPhs (MC2I), spindle-shaped MPhs (MC2II) and rod-shaped MPhs (MC2III). In vitro culture, on polymer samples and on cultural plastic, numerous MN CD -phenotypes differentiated in MPhs of 5 major morphotypes. It can be assumed, that each MPh-morphotype is a “product” of a specific line of differentiation of certain MN CD phenotypes. The morphological features of MPhs can determine differences in the functional activity of cells.

Rounded and elongated MPhs realize various types of motor activity onto the substrates through different adhesion molecules, and come from different subsets of MNs [26,27,28,29]. The amoeboid-like migration mode is characterized by rounded-shaped cells with a small number of short protrusions of the cytoplasm. In the mesenchymal migration mode, cells display an elongated shape with multiple long protrusions of the cell membrane [30]. Different intracellular signaling pathways have been associated with the amoeboid and mesenchymal cell migration modes. The amoeboid migration mode is ROCK-dependent [30].

**Figure 7:**
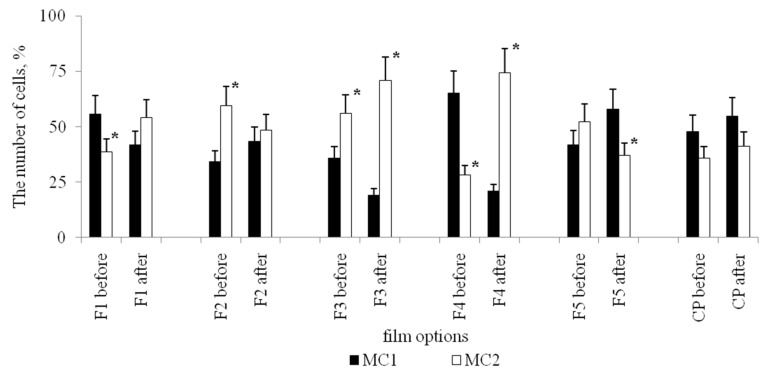
The number of rounded (MC1) and elongated (MC2) cells in the MPh population on PHA-films before and after stenting. CP—cultural plastic. F1–F5—PHA-films. *—The values for the variant after stenting, significantly different from the variant before stenting (*p* < 0.05).

Prior to stenting, elongated MPhs (MC2) dominating on films 1, 4, and 5 realizing the mesenchymal mode of locomotion (Figure 7). On films 2 and 3, roundedMPhs (MC1) with an amoeboid type of locomotion prevailed. So, the surface profiles of polymer films “determine” the type of cell locomotion, which is associated with certain rearrangements of the cytoskeleton and specific intracellular signaling.

As a criterion for comparison of the surface reliefs of PHA-nanofilms the parameter Rq, root-mean-square roughness, characterizing the height of relief, as one of the important topographical parameters, was used. Rq is programming cell behavior, such as the activity of proliferation, differentiation processes and mode of cellular locomotion [31,32].

**Figure 8:**
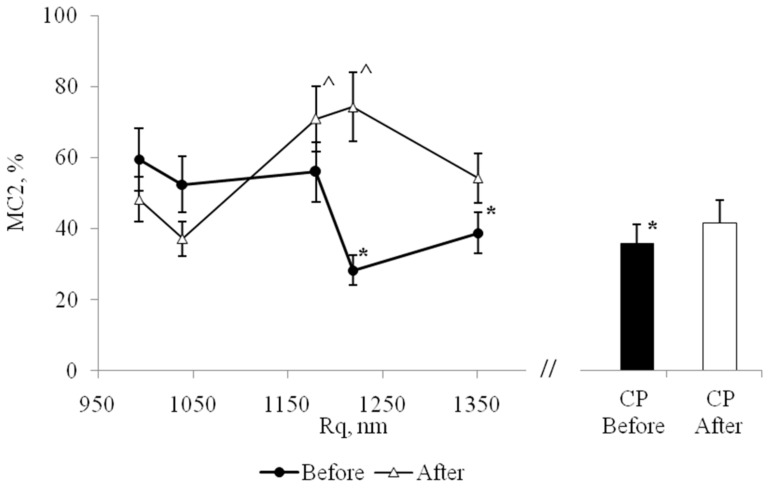
The dependence of the number of elongated MPhs (MC2) on the value of Rq, before (Before) and after (After) stenting. CP—cultural plastic. *—significant difference from the value for the first point, Rq_min_ = 993 nm, option before stenting, (*p* < 0,05). ^—significant difference from the value for the first point, Rq_min_ = 993 nm, option after stenting, (*p* < 0.05).

In this regard, we analyzed how Rq affects the quantity of elongated MPhs (MC2). Before stenting number of MPhs with mesenchymal locomotion onto the low reliefs was higher, than on higher reliefs (Figure 8), but this dependence has non-linear nature.

After stenting the ratio of the numbers of rounded and elongated forms, MC1/MC2, of MPhs significantly changed on all PHA-films (not as in the control). Thus, the “biological activity” of surface relief also depends on the initial functional state of the cells. After stenting, the quantitative ratios between different CD -phenotypes in the population of isolated MNs changed, which in culture in vitro led to changes in the ratios between different MPh -phenotypes. After stenting, a completely different dependence was formed between the value of Rq and number of elongated MPh (Figure 8): We noticed, that the abundance of elongated MPhs was higher on high reliefs, than on low reliefs. Also, before stenting, the high relief decreased the activity of the mesenchymal type of MPh locomotion, and after stenting, the high relief, on the contrary, increased the activity of the mesenchymal type of locomotion.

A subpopulation of MPhs MC2 implementing the mesenchymal type of cell locomotion, was characterized by morphological heterogeneity, as it was described above. We have identified three morphotypes, in accordance which their elongation factor: Filiform cells (MC2I), spindle-shaped cells (MC2II) and rod-shaped cells (MC2III) (Figure 3). It should also be noted that the magnitude of the elongation factor also determined the degree of “elongation” of the nucleus along the long axis of the cell. (Figure 3f).

Cell elongation is accompanied by cytoskeleton rearrangements, tension forces in the cytoskeleton system determine deformations (flattening and stretching in length) of the nucleus during the process of cell elongation [33]. It is known that deformations of the cell nucleus induce epigenome rearrangements through the system of mechanochemical signaling. This fact suggests that the identified cell morphotypes, differing by the elongation factor, are different epigenomic variants of mobile MPhs. In addition, the “lower side” of the nucleus repeats the profile of the substrates nanoreliefs, on which the cell “lies” [34,35]. Therefore, these morphological deformations of the nucleus can also trigger epigenomic rearrangements in it. The combination of these two types of nuclear deformation, flattening—stretching in length, and copying of nanoscale elements of surface relief, can determine the variability of epigenomic MPh variants on different PHA films.

The subpopulation of MPhs, realizing amoeboid-like locomotion, MC1, was represented by 2 cell morphotypes: Rounded mononucleated, MC1II, and rounded multinucleated, MC1I, cells. The ratio MC1I/MC1II varied significantly on different samples, both before and after stenting.

**Figure 9:**
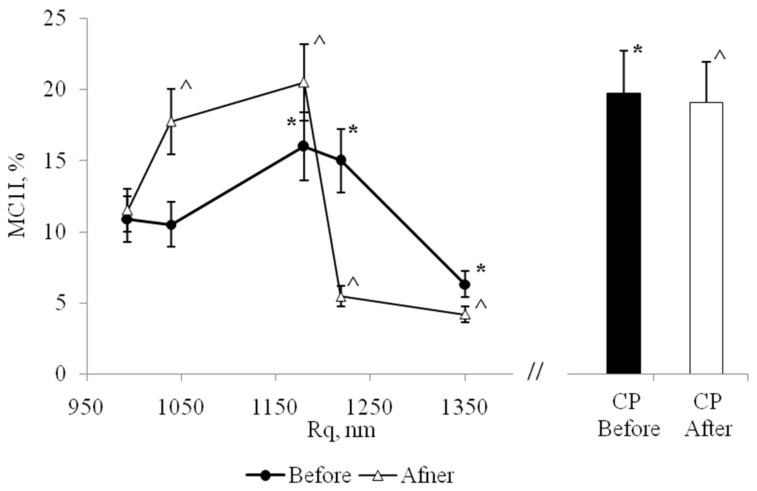
The dependence of the number of multinucleated MPhs (MC1I) on the value of Rq before (Before) and after (After) stenting. CP—cultural plastic. *—significant difference from the value for the first point, Rq_min_ = 993 nm, option before stenting, (*p* < 0.05).^—significant difference from the value for the first point, Rq_min_ = 993 nm, option after stenting, (*p* < 0.05).

Big multinucleated MPhs MC1I are the result of a fusion of several mononucleated cells. The fusion process is a cellular MPh-reaction, which is triggered, in particular, as a result of interaction with surfaces with a large radius of curvature.An example is multinucleated osteoclasts, MPhs of bone tissue, which “work” with this type of surfaces [36,37].Cultural plastic is characterized by a large radius of surface curvature, and high cell fusion activity could be expected here.However, in some variants (film 1, before and after stenting; film 5 after stenting), the number of MC1I was the same, as in cultural plastic. An increase in the height of the relief was accompanied by a decreasing in a number of MC1I (Figure 9). For the option “after stenting”, the diminishing of MC1I on high reliefs was much greater, than for the option “before stenting” (Figure 9).

The formation of multinucleated MPhs is the adaptation, aimed at developing the degradation of materials with a large radius of surface curvature [38]. The number of multinucleated MPhs on bionanofilms can be considered as a criterion of materials’ biodegradation activity.We discovered that high-relief substrates are more resistant to biodegradation: The activity of fusion and formation of multinucleated MPhs on such substrates were low, especially for the “after stenting” option. These results demonstrate, that the PHA-biodegradation depends on its surface topography, and on cells functional status.PHAs films 3 and 4 were the most stable: After the stenting, the number of multinucleated MPhs on these samples was the lowest.

In vitro the ratio between the rounded and elongated MPhs can be used for estimation of the M1/M2 polarization processes: Rounded MPhs referred to as M1-phenotype (pro-inflammatory MPhs) and elongated MPhs as M2-phenotype (anti-inflammatory MPhs, involved in repair processes) [39,40]. Therefore, the revealed variability in the numbers of these morphological classes on different films can be associated with the influence of surface reliefs on the dynamics of polarization processes.

Along with the morphological parameters, the direction of MPh polarization processes was evaluated by the activity of production of marker cytokines. Increased production of IL-6 is considered as evidence of M1-polarization of MPhs. In the culture of MPhs, obtained after stenting, on all films and in cultural plastic, the activity of IL-6 production increased significantly, compared to the same experimental options before stenting. So, the activity of M1-MPh polarization on the same surface relief in vitro depends on the initial functional state of the cell population, the ratio of various CD -phenotypes. After stenting on sample 5, the production of IL-6 was the lowest (Figure 6a). The activity of IL-6 production did not correlate obviously with the absolute number of rounded MPhs, which are commonly referred to as M1-MPhs. (It should be noted, that in our experiments the inductors of M1-polarization were not used, and the identified effects are determined only by the surface relief). The morphological criterion of the M1-polarization is not universal, even for cell lines of MPhs on culture plastic. It is obvious that MN-MPhs, isolated from the blood of patients with AS, on complex surface reliefs realized diverse cellular reactions.

The levels of production of IL-6 was highest in samples 2 and 4, but we did not found correlations with the nano-profiles or molecular weight of samples, so we may assume, that presence of 3HB-monomers in large amount in the polymer may provoke a inflammative formation phenotype of MPhs in AS in vitro.

As for the PHA of different compositions in the investigation, the biological value of materials from this chemical group in their natural origin, good technical properties, high biocompatibility and full biodegradability, and however, there are contradictorily communications, reports on the influence of some monomers on the biocompatibility of products from these materials. In our investigation, we took the presence of manifested atherosclerosis as a factor, significant for the formation of reactive changes in response to stimulation by different nano-profiles of the samples, and the obtained data, scatter of their values do not allow us to draw unambiguous conclusions about the role of any of the monomers in this case, but we noted the presence of reliable features characteristic of each of the monomers that form the PHA-molecule. It should be emphasized, that the heterogeneity of reactions to multi-component PHA samples is probably supposed an extension of studies with the special attention to each of the monomers; however, the process of biosynthesis of PHA by *Cupriaviduseu throphus* B10646 is fraught with certain technical limitations. The biosynthesis of multi-component PHA is realized under limited cells nutrition with the addition of precursors compounds, which are in use by PHA-synthase during the construction of intracellular PHA-macromolecules. Usually, multi-component samples of PHA obtained with an excess of various types of precursors in the culture medium at different periods of the synthesis process, in order to reveal the breadth of activity of PHA-synthase as a non-specific polymerase, and enzyme activity is determined by analysis of monomer composition of products. The analysis of the activity of nano-reliefs of all the monomers, involved in the current study, in separate—is a task of the next level of complexity.

## 5. Conclusions

MN-MPhs are cells, who are, among others, interacting with the implanted material in the body, and forms a local molecular microenvironment. This microenvironment determines the success of implantation efforts: Either the proliferation and differentiation of cells are activated, and the structure and functions of the tissue are restored, or a zone of local inflammation is formed, and degenerative processes and fibrosis are continued. Through the formation of a certain micro-nanorelief on the surface of the implant, one can modify the relationships of the MPhs with the implant, and “encode” their behavior. The choice of MPhs is important in screening the biological activity of the surface topography of potential implants. MPhs, obtained from transformed cell lines and MPhs of healthy donors are convenient model systems, but their behavior on the same reliefs undoubtedly will differ significantly from the behavior of MPhs that differentiate from blood MNs of patients with atherosclerosis. Therefore, adequate screening of the “biological activity” of surface reliefs for coatings materials of vascular stents for patients with atherosclerosis is possible only when using MN-MPhs, obtained from the blood of such patients.

Developed system of estimation of functional activity of MN-MPh cells of AS-patients is valid for the evaluation of the activity of nano-reliefs of biodegradable bionanomaterials towards cells, mainly responsible for the faith of AS-plaque. The process of stenting obviously triggered cascades of cell reactions, potentially causing the exacerbation of inflammation and re-stenosis. We showed the possibility of adequate reproduction of the regulatory influence of surface topography of implant on the dynamics of local MN-MPh differentiation processes in M1/M2 phenotypes, and activity of pro-inflammatory or regenerative events in vitro against the background of atherosclerosis. As a criterion of the formation of M1 or M2 phenotypes in the culture of MN-MPhs we used the activity of the production with cells IL-6 and IL-10, as confirmed in much research, CD antigenic spectrum of cells, and cell morphology, what is not so distinct. We got massive of data by five compounds of naturally-produced PHAs, as a perspective material for the development of implants and their coatings for AS-patients, but it is still prematurely to draw definitive conclusions about the most suitable compositions of them. As a more valuable observation, we indicate reliable alterations of the levels of measured cytokines and specific morphological transformations of cells on PHA-substrates with different surface topography. In addition, this model system can be useful for assessing the biodegradation activity of materials in vitro: The cell fusion activity and formation of multinucleated MPhs significantly varied, depending on the features of the surface topography.

## Figures and Tables

**Table 1:** Functions of CD antigens [23].

CDAG	Expression	Functions CDAG
CD14	MNs, MPhs	Receptor for complex of lipopolysaccharides and lipopolysaccharide binding protein
CD16	MPhs	Low affinity Fc receptor. Mediates phagocytosis and the antibody-dependent cellular cytotoxicity.
CD31	MNs	Cell adhesion. Plays a key role in leukocyte trafficking across the endothelium.
CD36	MNs, MPhs	Scavenger receptor preferentially found within lipid rafts. Mediatesadhesion and phagocytosis
CD62L	MNs	Leukocyte rolling and homing on activated endothelium.
CD68	MNs, MPhs	Possibly has a role in phagocytic activities of tissue macrophages, both in intracellular lysosomal metabolism and extracellular cell-cell and cell-pathogen interactions. Binds to tissue- and organ-specific lectins or selectins, allowing homing of macrophage subsets to particular sites. May allow macrophages to crawl over selectin bearing substrates or other cells.
CD163	MNs, MPhs	Clearance and endocytosis of hemoglobin/haptoglobin complexes by macrophages.
CD206	MNs, MPhs	Endocytosis (phagocytosis and pinocytosis) of mannose-containing solutes.

## References

[1] Loye A.M., Kinser E.R., Bensouda S., Shayan M., Davis R., Wang R., Chen Z., Schwarz U.D., Schroers J., Kyriakides T.R. (2018). Regulation of Mesenchymal Stem Cell Differentiation by Nanopatterning of Bulk Metallic Glass. Sci. Rep..

[2] Su N., Gao P.-L., Wang K., Wang J.-Y., Zhong Y., Luo Y. (2017). Fibrous scaffolds potentiate the paracrine function of mesenchymal stem cells: A new dimension in cell-material interaction. Biomaterials.

[3] De Peppo G.M., Agheli H., Karlsson C., Ekström K., Brisby H., Lennerås M., Gustafsson S., Sjövall P., Johansson A., Olsson E. (2014). Osteogenic response of human mesenchymal stem cells to well-defined nanoscale topography in vitro. Int. J. Nanomed..

[4] Park J., Bauer S., Schlegel K.A., Neukam F.W., Von Der Mark K., Schmuki P. (2009). TiO2Nanotube Surfaces: 15 nm-An Optimal Length Scale of Surface Topography for Cell Adhesion and Differentiation. Small.

[5] Yamada M., Kato E., Yamamoto A., Sakurai K. (2016). A titanium surface with nano-ordered spikes and pores enhances human dermal fibroblastic extracellular matrix production and integration of collagen fibers. Biomed. Mater..

[6] Schvartzman M., Palma M., Sable J., Abramson J., Hu X., Sheetz M.P., Wind S.J. (2011). Nanolithographic Control of the Spatial Organization of Cellular Adhesion Receptors at the Single-Molecule Level. Nano Lett..

[7] Khang N. (2015). Real time macrophage migration analysis and associated pro-inflammatory cytokine release on transparent carbon nanotube/polymer composite nano-film. Nanotechnology.

[8] Huang J., Grater S.V., Corbellini F., Rinck S., Bock E., Kemkemer R., Kessler H., Ding J., Spatz J.P., Rinck-Jahnke S. (2009). Impact of Order and Disorder in RGD Nanopatterns on Cell Adhesion. Nano Lett..

[9] Hulshof F.F., Zhao Y., Vasilevich A., Beijer N.R., De Boer M., Papenburg B.J., Van Blitterswijk C., Stamatialis D., De Boer J. (2017). NanoTopoChip: High-throughput nanotopographical cell instruction. Acta Biomater..

[10] Treiser M.D., Yang E.H., Gordonov S., Cohen D.M., Androulakis I.P., Kohn J., Chen C.S., Moghe P.V. (2010). Cytoskeleton-based forecasting of stem cell lineage fates. Proc. Natl. Acad. Sci. USA.

[11] Maniotis A.J., Chen C.S., Ingber D.E. (1997). Demonstration of mechanical connections between integrins, cytoskeletal filaments, and nucleoplasm that stabilize nuclear structure. Proc. Natl. Acad. Sci. USA.

[12] Ahn E.H., Kim Y., An S.S., Ahn E.H., Afzal J., Lee S., Kwak M., Suh K.-Y., Kim D.-H., Kshitiz (2014). Spatial control of adult stem cell fate using nanotopographic cues. Biomaterials.

[13] Volova T.G., Vinogradova O.N., Zhila N.O., Kiselev E.G., Peterson I.V., Vasil’Ev A.D., Sukovatyi A.G., Shishatskaya E.I. (2017). Physicochemical properties of multicomponent polyhydroxyalkanoates: Novel aspects. Polym. Sci. Ser. A.

[14] Berg K.E., Ljungcrantz I., Andersson L., Bryngelsson C., Hedblad B., Fredrikson G.N., Nilsson J., Björkbacka H. (2012). CD14++CD16- monocytes predict cardiovascular events. Circ. Cardiovasc. Genet..

[15] Carlin L.M., Stamatiades E.G., Auffray C., Hanna R.N., Glover L., Vizcay-Barrena G., Hedrick C.C., Cook H.T., Diebold S., Geissmann F. (2013). Nr4a1-dependent Ly6C(low) monocytes monitor endothelial cells and orchestrate their disposal. Cell.

[16] Cros J., Cagnard N., Woollard K., Patey N., Zhang S.-Y., Senechal B., Puel A., Biswas S.K., Moshous D., Picard C. (2010). Human CD14dim monocytes patrol and sense nucleic acids and viruses via TLR7 and TLR8 receptors. Immunity.

[17] Hanna R.N., Cekic C., Sag D., Tacke R., Thomas G.D., Nowyhed H., Herrley E., Rasquinha N., McArdle S., Wu R. (2015). Patrolling monocytes control tumor metastasis to the lung. Science.

[18] Rossol M., Kraus S., Pierer M., Baerwald C., Wagner U. (2012). The CD14(bright) CD16+ monocyte subset is expanded in rheumatoid arthritis and promotes expansion of the Th17 cell population. Arthritis Rheum..

[19] Rogacev K.S., Cremers B., Zawada A.M., Seiler S., Binder N., Ege P., Grobe-Dunker G., Heisel I., Hornof F., Jeken J. (2012). CD14++CD16+ monocytes independently predict cardiovascular events: A cohort study of 951 patients referred for elective coronary angiography. J. Am. Coll. Cardiol..

[20] Urra X., Villamor N., Amaro S., Gómez-Choco M., Obach V., Oleaga L., Planas A.M., Chamorro A. (2009). Monocyte Subtypes Predict Clinical Course and Prognosis in Human Stroke. J. Pharmacol..

[21] Azeredo E.L., Neves-Souza P.C., Alvarenga A.R., Reis S.R.N.I., Torrentes-Carvalho A., Zagne S.O., Nogueira R.M.R., Oliveira-Pinto L.M., Kubelka C.F. (2010). Differential regulation of toll-like receptor-2, toll-like receptor-4, CD16 and human leucocyte antigen-DR on peripheral blood monocytes during mild and severe dengue fever. Immunology.

[22] Hamers A.A., Dinh H.Q., Thomas G.D., Marcovecchio P., Blatchley A., Nakao C.S., Kim C., McSkimming C., Taylor A.M., Nguyen A.T. (2019). Human Monocyte Heterogeneity as Revealed by High-Dimensional Mass Cytometry. Arterioscler. Thromb. Vasc. Biol..

[23] Abcam. https://docs.abcam.com/pdf/immunology/Guide-to-human-CD-antigens.pdf.

[24] Yang J., Zhang L., Yu C., Yang X.-F., Wang H. (2014). Monocyte and macrophage differentiation: Circulation inflammatory monocyte as biomarker for inflammatory diseases. Biomark. Res..

[25] Wildgruber M., Aschenbrenner T., Wendorff H., Czubba M., Glinzer A., Haller B., Schiemann M., Zimmermann A., Berger H., Eckstein H.H. (2016). The “intermediate” CD14^++^CD16^+^ monocyte subset increases in severe peripheral artery disease in humans. Sci. Rep..

[26] Lee H.S., Stachelek S.J., Tomczyk N., Finley M.J., Composto R.J., Eckmann D.M. (2013). Correlating macrophage morphology and cytokine pro¬duction resulting from biomaterial contact. J. Biomed. Mater. Res. A.

[27] Hind L.E., Dembo M., Hammer D.A. (2015). Macrophage motility is driven by frontal-towing with a force magnitude dependent on substrate stiffness. Integr. Boil..

[28] Lu J., Webster T.J. (2015). Reduced immune cell responses on nano and submicron rough titanium. Acta Biomater..

[29] Collison J.L., Carlin L.M., Eichmann M., Geissmann F., Peakman M. (2015). Heterogeneity in the Locomotory Behavior of Human Monocyte Subsets over Human Vascular Endothelium In Vitro. J. Immunol..

[30] Van Goethem E., Poincloux R., Gauffre F., Maridonneau-Parini I., Le Cabec V. (2010). Matrix architecture dictates three-dimensional migration modes of human macrophages: Differential involvement of proteases and podosome-like structures. J. Immunol..

[31] Yokose S., Klokkevold P.R., Takei H.H., Kadokura H., Kikui T., Hibino Y., Shigeta H., Nakajima H., Kawazu H. (2018). Effects of surface microtopography of titanium disks on cell proliferation and differentiation of osteoblast-like cells isolated from rat calvariae. Dent. Mater. J..

[32] Czeisler C., Short A., Nelson T., Gygli P., Ortiz C., Catacutan F.P., Stocker B., Cronin J., Lannutti J., Winter J. (2016). Surface topography during neural stem cell differentiation regulates cell migration and cell morphology. J. Comp. Neurol..

[33] Hulsman M., Hulshof F., Unadkat H., Papenburg B.J., Stamatialis D.F., Truckenmüller R., Van Blitterswijk C., De Boer J., Reinders M.J., Stamatialis D. (2015). Analysis of high-throughput screening reveals the effect of surface topographies on cellular morphology. Acta Biomater..

[34] Wang K., Bruce A., Mezan R., Kadiyala A., Wang L., Dawson J., Rojanasakul Y., Yang Y. (2016). Nanotopographical Modulation of Cell Function through Nuclear Deformation. ACS Appl. Mater. Interfaces.

[35] Hanson L., Zhao W., Lou H.-Y., Lin Z.C., Lee S.W., Chowdary P., Cui Y., Cui B. (2015). Vertical nanopillars for in situ probing of nuclear mechanics in adherent cells. Nat. Nanotechnol..

[36] Fais S., Burgio V.L., Silvestri M., Capobianchi M.R., Pacchiarotti A., Pallone F. (1994). Multinucleated giant cells generation induced by interferon-gamma. Changes in the expression and distribution of the intercellular adhesion molecule-1 during macrophages fusion and multinucleated giant cell formation. Lab. Investig..

[37] Helming L., Winter J., Gordon S. (2009). The scavenger receptor CD36 plays a role in cytokine-induced macrophage fusion. J. Cell Sci..

[38] Miron R.J., Bosshardt D.D. (2017). Multinucleated giant cells: Good guys or bad guys. Tissue Eng. Part B Rev..

[39] Rey-Giraud F., Hafner M., Ries C.H. (2012). In Vitro Generation of Monocyte-Derived Macrophages under Serum-Free Conditions Improves Their Tumor Promoting Functions. PLoS ONE.

[40] Heinrich F., Lehmbecker A., Raddatz B.B., Kegler K., Tipold A., Stein V.M., Kalkuhl A., Deschl U., Baumgärtner W., Ulrich R. (2017). Morphologic, phenotypic, and transcriptomic characterization of classically and alternatively activated canine blood-derived macrophages in vitro. PLoS ONE.

